# Dietary Changes over 25 Years in Tianjin Residents: Findings from the 1986–1988, 2000–2004, and 2008–2011 Nutrition Surveys

**DOI:** 10.3390/nu8020062

**Published:** 2016-01-22

**Authors:** Xuan Wang, Yuntang Wu, Xumei Zhang, Meilin Zhang, Guowei Huang

**Affiliations:** Department of Nutrition and Food Science, School of Public Health, Tianjin Medical University, 22 Qixiangtai Road, Heping District, 300070 Tianjin, China; wangxuan@tmu.edu.cn (X.W.); wuyuntang@tmu.edu.cn (Y.W.); zhangxumei@tmu.edu.cn (X.Z.); defjmmm@163.com (M.Z.)

**Keywords:** nutrition survey, dietary pattern, urban area, rural area

## Abstract

In China, the rates of chronic diseases characteristic of countries in nutritional transition have been increasing. However, few studies have examined diet changes in recent decades. We analyzed dietary changes in Tianjin, China. The data in this descriptive, population-based study in ≥18-year-old adults were collected from three surveys from 1986 to 2011. Food consumption and nutrient intake were compared among the three surveys separately for urban and rural areas. Differences in food consumption between urban and rural areas in different periods were also shown. The consumption of cereals, vegetables, and oils decreased, and that of fruits and beans increased in both urban and rural areas. Moreover, the total consumption of animal foods, especially milk, increased (0.01% in 1986–1988; 1.72% in 2008–2011) in rural areas. Although milk consumption also increased in urban areas, consumption of other animal foods decreased (19.33% in 1986–1988; 13.74% in 2008–2011). Meanwhile, cereals consumption rebounded from 22.63% in 2000–2004 to 29.75% in 2008–2011. Moreover, the lack of dairy products and some nutrients, e.g., retinol, calcium, and dietary fiber (<80% of recommended nutrient intake), in the diet persisted in both urban and rural areas. In conclusion, differences in diet between urban and rural areas decreased over time.

## 1. Introduction

Human survival has always been based on foods and nutrients, which are also important factors affecting the population’s quality and are closely related to economic and social development. Meanwhile, economic progress and high levels of education can also change people’s dietary patterns. Technological changes have reduced physical activity for work, travel, home production, and leisure. Chinese consumption patterns have been transformed by changes in food technology, import controls, food pricing, and mass media. China has undergone many marked dietary pattern shifts since 1949. These include: (1) a period (1949–1957) when food production was inadequate and cereal consumption was low; (2) the famine (1957–1962), which was linked with the Great Leap Forward; (3) a strong recovery (1962–1979); (4) the subsequent reform period (1979–1985) after the liberalization of food production, when the annual economic growth rate was greater than 10%; and (5) the current period (since 1985), in which continued rapid economic growth and a remarkable shift in diet structure has occurred [1,2,3,4]. Rapid economic and social change has transformed urban China and much of its rural sector as well [5].

The classic Chinese diet includes cereals and vegetables with few animal foods. It is a diet that many scholars consider to be the healthiest when adequate intake is achieved. An earlier study showed rapid changes in diet [6]. As the classic dietary pattern shifts, intakes of cereals and many lower-fat, mixed dishes are being replaced, animal foods are becoming popular, and the consumption of edible oils is increasing quickly. This shift in diet composition and corresponding body composition has been accompanied by many positive and some negative changes [2]. Although infectious diseases and hunger, the important causes of death in the 1950s, no longer affect most of the population, the mortality burden has shifted to diet-related, non-communicable diseases (DR-NCDs), with a rapid increase in the prevalence of obesity. Obesity and non-communicable diseases are the major causes of morbidity, disability, and mortality in China, and the health system has to develop in order to care for people with these conditions [7,8,9].

In 1985, China entered the fifth dietary pattern period (*i.e.*, the current period), during which the economy has further improved. China has been undergoing a fast shift towards a stage of nutrition transition dominated by a high intake of fat and animal foods by 2004, as well as a high prevalence of DR-NCDs [10]. Because of these, the Ministry of Health consigned the Chinese Nutrition Society constituting the “Chinese Dietary Guidelines” (2007) and the “Food Guide Pagoda (FGP) for Chinese Residents” (2007) which were published after two years of hardships [11]. To our knowledge, no study based on the changes after that has been carried out in China to date. The present study added some new information after 2007, which had not been assessed before, and explored a nutritional shift in different time periods by using a sample from urban and rural Tianjin, one of four large cities in China. We used data on individual diets from three independent representative samples of the Tianjin population to examine the differences in food and nutrient intake and in the source of energy, protein and fat for the periods of 1986–1988, 2000–2004, and 2008–2011(contained several key time points of dietary changes during these periods). Moreover, we compared the latest data with FGP (2007) to reveal the changes in 2008–2011. The full implications of these changes are then discussed.

## 2. Experimental Section

### 2.1. Data Sources and Subjects

Data from urban and rural areas were analyzed respectively in this study because of the imbalance in economic development between urban and rural areas in China. Data for this study were primarily obtained from three sources. First, the nutrition survey data from 1986 to 1988 in urban and rural Tianjin (S1); second, annual household consumption surveys data from 2000 to 2004 (S2); and the third, nutrition survey data from 2008 to 2011 in urban and rural Tianjin (S3). The three surveys covered all six districts from urban areas and all five counties from rural areas in Tianjin. Data from 1986 to 1988 and from 2000 to 2004 were partially available [12,13,14]. The use of the data for 2008–2011 survey was approved by the ethics committee of Tianjin Medical University.

A multistage, random cluster sample was used to draw the sample in three surveys. The surveys collected information on all individuals, including all age groups, living in the household. Our study included adults aged at least 18 years (≥18 years) in three surveys to comprise the study population (Table 1). Moreover, the data for further comparison were converted to the level of reference person (an 18-year-old man with light physical activity weighing 60 kg) so that the results are consistent and comparable.

**Table 1:** Composition of study population in Tianjin by three surveys (*n*, %)

Population Group	1986–1988		2000–2004		2008–2011	
	Urban (%)	Rural (%)	Urban (%)	Rural (%)	Urban (%)	Rural (%)
Sex						
Male	7061 (48.5)	1603 (49.3)	8076 (49.1)	5426 (49.4)	4674 (47.2)	3737 (49.0)
Female	7488 (51.5)	1649 (50.7)	8385 (50.9)	5555 (50.6)	5222 (52.8)	3891 (51.0)
Total	14,549	3252	16,461	10,981	9896	7628
Age (years)						
18–29	3577 (24.6)	826 (25.3)	2316 (14.1)	2888 (26.3)	1017 (10.3)	1985 (26.0)
30–39	3334 (22.9)	760 (23.4)	4111 (25.0)	2712 (24.7)	2723 (27.5)	1840 (24.1)
40–49	2990 (20.6)	659 (20.3)	3137 (19.0)	2559 (23.3)	2672 (27.0)	1755 (23.0)
50–59	2464 (16.9)	650 (20.0)	3979 (24.2)	1787 (16.3)	1898 (19.2)	1419 (18.6)
60–	2184 (15.0)	357 (11.0)	2918 (17.7)	1035 (9.4)	1586 (16.0)	629 (8.3)
Total	14,549	3252	16,461	10,981	9896	7628

### 2.2. Dietary Analysis

**Figure 1:**
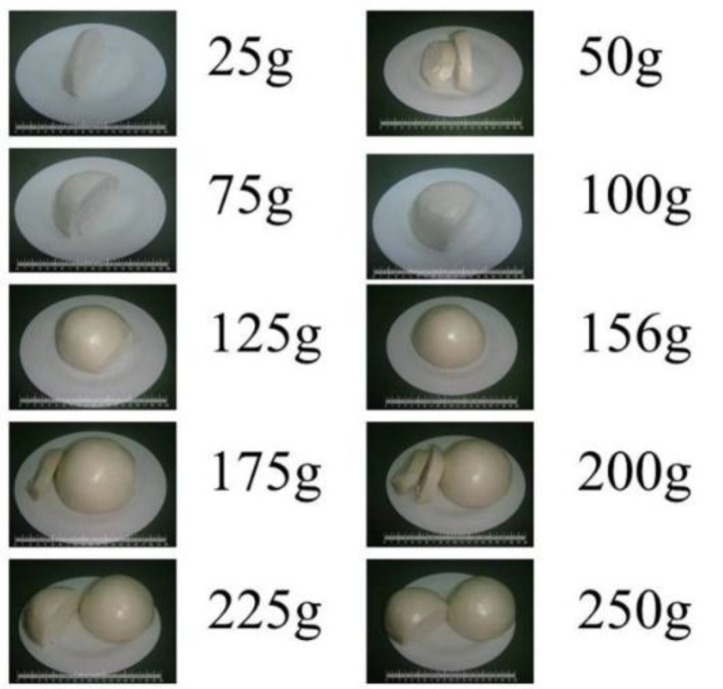
Example of selection pictures for aiding estimatation of the amount of steamed buns eaten.

Dietary data were collected via in-home interviews by a combination of the weighting method and a seven-day consecutive record method. A food-weighted record method was used for S1 and S2. The method was determined by weighing all food consumed by the household over seven consecutive randomly selected days. All remaining food was weighed and recorded at the beginning and the end of the survey. Household food consumption was determined by examining changes in every kind of food from the beginning to the end of each day. All the newly brought foods were recorded. Food eating outside was estimated when weighing was not possible. A seven-day consecutive record method was used in S3; every household member was asked to record all food consumed over the previous 24 h for each of the seven days, no matter where it was eaten. Individuals were asked to record the name and amounts of food consumed for every meal. The amount of food in each dish was estimated according to some food pictures which showed different amounts of food on a 20 cm paper plate (e.g., Figure 1). Compared to the food-weighted record method, food intakes assessed by 24 h recall were similar, and the relative differences were less than 10% of most food items [15]. Data obtained by these methods correlated well with respect to individual food items and calculated nutrients.

The food groups included were based on Chinese Food Composition Table [16]. The following twenty food groups were included: cereals and grain products; potatoes and starches; beans and their products; vegetables; mushrooms; fruits; nuts and seeds; red meat and their products; poultry and their products; fish and shellfish; milk and dairy products; egg and egg products; snack and cakes; fast food; drinks; alcohol; sugar; flavors; fats and oils. However, S3 for 2008–2011 did not include sugar, flavors, drinks and alcohol. In order to ensure the consistency of the food category and the readability of the information, we removed these four groups. According to the results of three surveys, food items with similar nutritional profiles were merged, and food items people ate less were put together into new group which was called others. In total, 12 sub-categories were summarized to compare the average food consumption. Moreover, 10 main food groups were used to compare the food proportion according to the Food Guide Pagoda [17].

Food and nutrient intakes were assessed by calculating the amount for each reference person per day according to the constitution of the population. A quantitative assessment of dietary intake was analyzed using a computer dietary analysis. We determined total calories; percentage of calories from total carbohydrate, protein, and fat; percentage of proteins from different kinds of foods; and the following nutrients: cholesterol, fiber, vitamin A (retinol), vitamin B1 (thiamine), vitamin B2 (riboflavin), niacin, vitamin C (ascorbic acid), calcium, iron, zinc, and selenium using the Chinese Food Composition Table [16]. The total retinol value of the diet was evaluated by the retinol equivalent (RE). Food intakes were compared with the levels in the 2002 Chinese National Nutrition and Health Survey (NNHS) [18,19] and the recommended intakes in the food guide pagoda (2007) [17], including intake of cereals (250–400 g/day), vegetables (300–500 g/day), fruits (200–400 g/day), meats and poultry (50–75 g/day), fish and seafood (50–100 g/day), eggs and egg products (25–50 g/day), milk and dairy products (300 g/day), beans (30–50 g/day), and oil (25–30 g/day). We also determined the percentage of selected recommended nutrient intakes (RNIs) and adequate intakes (AIs) [17]. The intake of vitamin A was compared using AI; total fat (≤30% kcal/day), cholesterol (≤300 mg/day), vitamin B1, vitamin B2, niacin, vitamin C, calcium, iron, zinc, and selenium were compared using RNI.

### 2.3. Statistical Analysis

The food and nutrient intake were calculated using Microsoft Office Excel 2003 (Microsoft Corp., Redmond, WA, USA). All analyses were performed separately for urban and rural areas. Mean daily nutrient intake was derived for descriptive purposes. Values were expressed as means ± SD. Nutrient intake and food consumption among the periods of 1986 to 1988, 2000 to 2004, and 2008 to 2011 were compared using an analysis of variance. The statistical analysis was performed with SPSS 13.0 for Windows (Chicago, IL, USA).

## 3. Results

### 3.1. Food Consumption Patterns

#### 3.1.1. Proportions for Different Food Groups

A reduction of proportions for cereals, vegetables, and oil and an increase of proportions for fruits, beans, milk and dairy products were found in both urban and rural areas for the periods of 1986–1988, 2000–2004, and 2008–2011 (Supplementary Table S1). Especially the proportions for fruits and milk and dairy products increased sharply (*i.e.*, from 1.01% for 1986–1988 to 16.29% for 2008–2011 and from 0.01% for 1986–1988 to 1.72% for 2008–2011, respectively) in rural areas, while the proportion for cereals decreased significantly. Whereas the proportions for cereals increased to 29.75% for 2008–2011 after falling to the lowest 22.63% for 2000–2004 in urban areas. Moreover, it also showed that the total proportion for animal foods except milk and dairy products decreased in urban areas and increased in rural areas.

#### 3.1.2. Average Intakes for Different Food Groups

The mean food consumption levels from 2002 NNHS and FDP (2007) were used as reference. The average intakes for different food groups for the periods of 1986–1988, 2000–2004, and 2008–2011 were compared to each other (Table 2). Furthermore, a comparison of differences between urban and rural areas in three periods was carried out for each food group (Figure S1).

##### Cereals

In urban areas, cereal intake decreased considerably from 1986–1988 to 2000–2004, but increased during the period of 2008–2011 (*p* < 0.001) (Table 2). In rural areas, the mean intake of cereals decreased gradually, from 483.03 g/reference person/day to 442.26 g/reference person/day (*p* = 0.807) (Table 2). Therefore, the shift in cereal intake for rural areas was not obvious among the three discrete stages from 1986 to 2011. The difference between urban and rural residents’ intakes increased from 88.36 g/reference person/day for 1986–1988 to 222.43 g/reference person/day for 2000–2004, and then decreased to 51.04 g/reference person/day for 2008–2011(Figure S1). Both urban and rural residents’ cereal and tubers products intakes became slightly higher than the levels of FGP (Table 2).

##### Vegetables and Fruits

Intake of vegetables increased significantly in both urban and rural areas from 1986–1988 to 2000–2004, but decreased slightly for 2008–2011(Table 2). Urban residents’ intake was higher than rural residents’. The difference between urban and rural residents’ intakes decreased from 68.84 g/reference person/day for 1986–1988 to 53.86 g/reference person/day for 2000–2004 and then to 9.73 g/reference person/day for 2008–2011 (Figure S1). Moreover, both urban and rural residents’ vegetable intake in 2008–2011 was just a little lower than the recommended level of FGP (Table 2). However, the mean intake of fruits increased considerably across all three time periods in both urban and rural areas (Table 2). The intake of fruits for 2008–2011 was 4.57-fold and 25.28-fold higher than that for 1986–1988 in urban and rural areas, respectively. The difference between urban and rural residents’ intake decreased to 1.31 g/reference person/day (Figure S1). Furthermore, both urban and rural residents’ fruit intake in 2008–2011 was in the recommended range of FGP (Table 2).

##### Animal Foods

The mean intake of total animal foods increased over time in both urban and rural areas. Rural residents’ intake showed a more significant shift (the mean intake of total animal foods increased by 106.77 g/reference person/day during the study period), especially with regard to the intake of milk and dairy products (Table 2). Although the intake of milk was still less than the level of FGP recommended, the intake for 2008–2011 was 176.31-fold higher than that for 1986–1988. Although the overall increase (6.85 g/reference person/day) in intake for urban residents was less than that for rural residents, urban residents’ daily intake of total animal foods was higher than that for rural residents across all periods from 1986 to 2011. However, the intake of total animal foods except milk and dairy products decreased in urban areas and increased in rural areas (Table 2). Urban residents’ intake of meat and eggs was higher than the level of FGP recommended, while intake of milk was lower than that level. Rural residents’ intake of egg in 2008–2011 was higher than the level of FGP recommended, while intake of fish and milk was far lower than that (Table 2).

##### Beans and Bean Products

The intake of beans decreased from 1986–1988 to 2000–2004, but then increased considerably for 2008–2011 in both urban and rural areas, and was even higher than the level of FGP (Table 2). The mean intake of beans decreased by 5.72 g/reference person/day for urban residents and by 2.09 g/reference person/day for rural residents, and then increased by 82.77 g/reference person/day and 55.30 g/reference person/day in urban and rural areas, respectively.

**Table 2:** Average food consumption of different dietary food groups for urban and rural residents in Tianjin in three different periods (*x* ± *s*, g/reference person/day).

Food Category	Urban				Rural				2002 NNHS		Food Guide ^a^
	1986–1988	2000–2004	2008–2011	*p* Value	1986–1988	2000–2004	2008–2011	*p* Value	Urban	Rural	in 2007
Plant foods											
Cereals and grain products	394.67 ± 41.97	242.06 ± 10.12 **	391.22 ± 40.77 ^##^	0.000	483.03 ± 34.95	464.49 ± 84.28	442.26 ± 31.41	0.807	366.1	415.3	250~400 ^b^
Potatoes and starches	39.03 ± 16.27	36.00 ± 2.29	29.92 ± 4.04	0.565	31.30 ± 6.9	33.33 ± 11.80	42.67 ± 13.72	0.523	31.9	56.2	---
Beans and their products	21.23 ± 5.49	15.51 ± 10.52	98.28 ± 24.80 **^##^	0.000	12.23 ± 2.56	10.14 ± 6.67	65.44 ± 20.60 **^##^	0.001	15.5	16.2	30–50
Vegetables	272.67 ± 58.52	374.62 ± 19.62 *	289.07 ± 82.72	0.011	203.83 ± 46.98	320.76 ± 66.8	279.34 ± 58.96	0.069	251.9	284.1	300–500
Fruits	48.27 ± 6.90	208.82 ± 14.10 **	220.49 ± 58.23 **	0.000	8.67 ± 6.40	89.45 ± 5.67 **	219.18 ± 56.38 **^##^	0.000	69.3	36.6	200–400
Vegetable oils	37.63 ± 7.50	24.10 ± 2.61 **	27.23 ± 4.63 *	0.018	17.00 ± 3.84	25.11 ± 2.60	29.76 ± 5.43 *	0.012	40.2	29.9	25–30 ^c^
others	56.90 ± 28.40	66.20 ± 11.39	89.15 ± 35.11	0.323	20.97 ± 6.73	10.61 ± 0.61	76.00 ± 20.23 **^##^	0.000	---	---	---
Animal foods											
Meats and poultry and their products	83.50 ± 2.34	96.21 ± 3.65	80.34 ± 14.04 ^#^	0.024	19.93 ± 4.84	32.14 ± 0.69 *	64.40 ± 8.51 **^##^	0.000	104.4	69.9	50–75
Fish and shellfish	62.50 ± 8.34	49.62 ± 12.67	48.58 ± 7.88	0.206	26.73 ± 8.36	23.13 ± 3.34	37.93 ± 7.18 ^#^	0.043	44.9	24.4	50–100
Egg and egg products	72.43 ± 11.96	54.43 ± 3.65 *	65.53 ± 20.51 *	0.018	29.87 ± 5.76	38.53 ± 8.14	60.72 ± 23.70	0.062	33.2	19.9	25–50
Milk and dairy products	44.00 ± 6.71	61.32 ± 13.39	74.19 ± 2.78 ^#^	0.045	0.13 ± 0.06	7.20 ± 9.32	22.92 ± 10.02 *	0.046	65.8	11.2	300
Animal oils and fats	0.70 ± 0.17	0.08 ± 0.05 **	1.34 ± 0.84 ^##^	0.007	6.47 ± 2.27	2.31 ± 0.73 *	3.93 ± 0.97*	0.013	3.8	10.5	---
Total	1133.53 ± 62.02	1228.97 ± 70.48	1415.34 ± 44.18 **	0.006	860.16 ± 12.17	1057. 20 ± 39.56 *	1344.55 ± 60.54 **^#^	0.000	---	---	---

^a^: from the Chinese food guide pagoda in 2007; ^b^: both from cereals and tubers products; ^c^: total oil(both from animal and vegetable); * *p* < 0.05: *vs.* 1986–1988; ** *p* < 0.01: *vs.* 1986–1988; ^#^ *p* < 0.05: *vs.* 2000–2004; ^##^ *p* < 0.01: *vs.* 2000–2004; Abbreviation: 2002 NNHS: 2002 Chinese National Nutrition and Health Survey.

##### Oils

The shift in oil intake in both urban and rural residents is very interesting. Urban residents’ mean intake of oils was higher than that for rural residents for 1986–1988. Urban residents’ mean intake of oils decreased by 14.15 g/reference person/day for 2000–2004, while rural residents’ mean intake of oils increased by 3.95 g/reference person/day. Then, both urban and rural residents’ intake increased for 2008–2011. Overall, rural residents’ mean intake of oils came from behind and was higher than that for urban residents, and was even higher than the level of FGP recommended (Table 2).

### 3.2. Caloric and Nutrient Intakes

#### 3.2.1. Caloric Intakes

Urban residents’ mean caloric intakes decreased over time, while there were no big changes in rural areas (Table 3). Urban residents’ mean caloric intakes were higher than rural residents’ intakes for 1986–1988. During the five-year period from 2000 to 2004, urban residents’ mean caloric intakes decreased by 671.46 kcal/reference person/day, while rural residents’ increased by 131.13 kcal/reference person/day. The difference between urban and rural residents’ caloric intakes increased to 444.29 kcal/reference person/day by 2000–2004. Then, urban residents’ intakes increased (by 164.84 kcal/reference person/day) and rural residents’ decreased (by 141.41 kcal/reference person/day) for 2008–2011. Finally, rural residents’ intakes were slightly higher than those of urban residents. The difference between urban and rural residents’ caloric intakes decreased to 138.04 kcal/reference person/day by 2008–2011(Table 3).

#### 3.2.2. Nutrients Intakes and Selected Nutrients Intakes as Percent of RNI or AI

Urban residents’ protein, fat, and carbohydrate intakes all decreased over time. Both protein and fat intakes increased in rural areas, but carbohydrate intake slightly decreased (Table 3). The intake of dietary fiber increased for both urban and rural residents. And it had increased by 185.24% in urban areas and by 249.34% in rural areas by 2008–2011 (Table 3). Rural residents’ mean intake of cholesterol increased more quickly than did urban residents’ (135.61% *vs.* 4.82%, respectively), and the cholesterol intake for rural residents was closer to that of urban residents for 2008–2011 (Table 3). Additionally, retinol significantly decreased among urban residents for both 2000–2004 and 2008–2011 (*p* < 0.01), riboflavin and niacin significantly increased among rural residents for 2008–2011, and thiamine significantly decreased for all residents and calcium and selenium significantly increased among rural residents for both 2000–2004 and 2008–2011 (Table 3).

### 3.3. Composition of Energy, Protein and Fat

**Figure 2:**
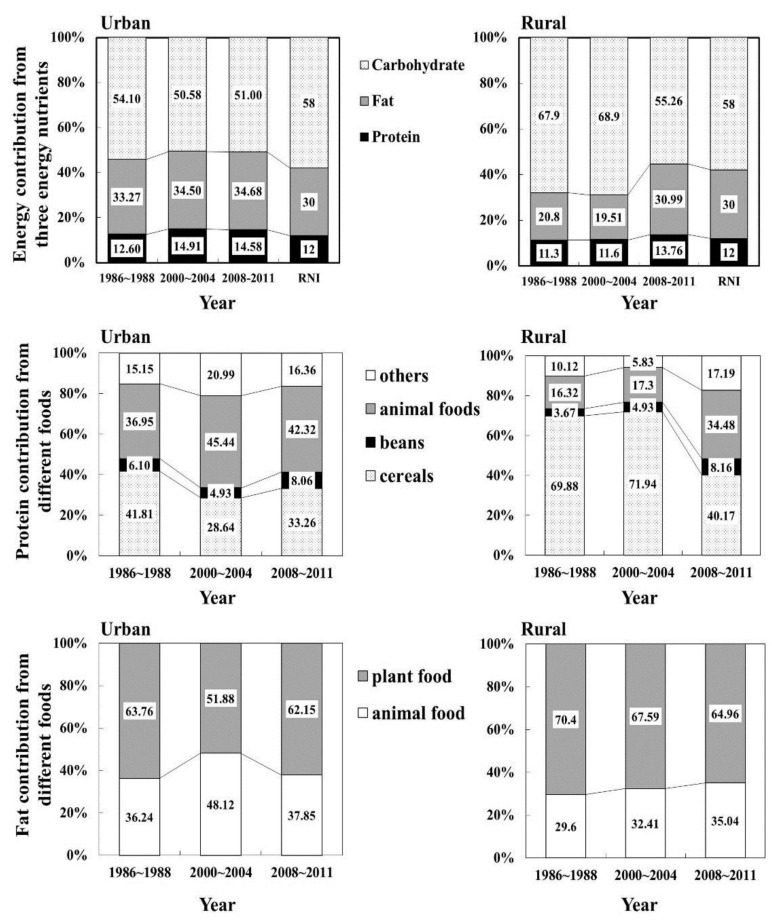
Composition of energy from three energy nutrients and compositions of protein and fat from different foods for urban and rural residents in three different periods.

Regarding the food sources for energy, energy from cereals and grain products decreased remarkably and that from animal foods and others increased significantly in rural areas in this study period (Table 4). In urban areas, after an increased proportion of animal foods, the proportion fell to 20.73% for 2008–2011 from 24.54% for 2000–2004. Furthermore, an increasing trend in energy from beans and their products and a decreasing trend in that from pure energy foods were found in both urban and rural areas (Table 4). Nutrient sources of energy changed remarkably: energy from carbohydrates decreased from 54.10% to 51.00% (lower than the RNI) in urban areas and from 67.9% to 55.26% (similar to the RNI) in rural areas, whereas energy from both protein and fat increased both in urban and rural areas (Figure 2). And these changes were more obvious in rural areas.

Among the energy components, there was little change in the proportion of energy from protein, but the proportion of protein from animals increased while protein from cereals decreased considerably. This change was especially remarkable for rural residents; only 16.32% of their protein came from animal foods for 1986–1988, but that increased to 17.3% for 2000–2004 and to 34.48% for 2008–2011, while protein from cereals decreased from 69.88% to 40.17% (Figure 2).

**Table 3:** Average energy and nutrients intake (percentage of RNI/AI) for urban and rural residents in Tianjin in three different periods (*x* ± s, /reference person/day)

Energy/Nutrients	Urban				Rural				RNI/AI
	1986–1988	2000–2004	2008–2011	*p* Value	1986–1988	2000–2004	2008–2011	*p* Value	
	(%)	(%)	(%)		(%)	(%)	(%)		
Energy (kcal)	2649.07 ± 272.18	1977.61 ± 92.86 **	2142.45 ± 156.31 *	0.003	2290.77 ± 71.12	2421.90 ± 96.30	2280.49 ± 38.91	0.098	2400
	(110.38)	(82.40)	(89.25)		(95.45)	(100.91)	(95.02)		
Protein (g)	83.63 ± 7.07	73.81 ± 4.97	78.08 ± 1.05	0.104	64.60 ± 2.31	70.26 ± 4.29	78.41 ± 0.58 **	0.010	75
	(111.51)	(98.41)	(104.11)		(86.13)	(93.68)	(104.55)		
Fat (g)	98.20 ± 13.95	75.91 ± 4.85 *	81.21 ± 5.16	0.025	52.83 ± 4.25	52.47 ± 2.34	78.43 ± 9.09 **^##^	0.001	
Carbohydrate (g)	357.57 ± 33.42	250.19 ± 10.37 **	275.02 ± 26.11 *	0.001	389.87 ± 25.54	417.45 ± 20.00	315.24 ± 30.77 *^##^	0.004	
Dietary fiber (g)	6.03 ± 0.55	10.13 ± 0.50	17.20 ± 7.95 **^#^	0.015	5.27 ± 0.32	16.24 ± 2.58 **	18.41 ± 7.82 **^##^	0.006	25
	(24.12)	(40.52)	(68.80)		(21.08)	(64.96)	(73.64)		
Retinol Equiv. (μg)	1377.00 ± 136.54	444.38 ± 122.22 **	624.15 ± 27.10 **	0.000	590.20 ± 73.72	426.469 ± 153.89	576.24 ± 94.85	0.217	800
	(172.13)	(55.55)	(78.02)		(73.78)	(53.31)	(72.03)		
Thiamine (mg)	1.93 ± 0.40	1.01 ± 0.08	1.10 ± 0.10	0.002	2.40 ± 0.17	1.44 ± 0.14 **	1.12 ± 0.07 **	0.000	1.4
	(137.86)	(72.14)	(78.57)		(171.43)	(102.86)	(80.00)		
Riboflavin (mg)	1.13 ± 0.12	1.10 ± 0.08 **	1.16 ± 0.17 *	0.744	0.73 ± 0.06	0.75 ± 0.04	1.14 ± 0.20 **^##^	0.002	1.4
	(80.71)	(78.57)	(82.86)		(52.14)	(53.57)	(81.43)		
Niacin (mg)	15.33 ± 0.85	18.05 ± 0.72 **	17.03 ± 0.22	0.004	14.40 ± 0.46	15.09 ± 0.76	16.91 ± 0.39 **^#^	0.010	14
	(109.5)	(128.93)	(121.64)		(102.86)	(107.79)	(120.78)		
Ascorbic acid (mg)	94.00 ± 21.17	165.43 ± 45.41	101.33 ± 0.03	0.055	58.13 ± 11.02	85.20 ± 24.19	95.29 ± 8.57	0.137	100
	(94.00)	(165.43)	(101.33)		(58.13)	(85.2)	(95.29)		
Calcium (mg)	475.1 ± 67.05	611.25 ± 92.44	625.15 ± 57.97	0.936	406.83 ± 30.03	482.40 ± 34.29 *	504.86 ± 97.01 **^##^	0.000	800
	(59.39)	(76.41)	(78.14)		(50.85)	(60.3)	(63.11)		
Iron (mg)	28.03 ± 2.80	24.20 ± 1.24	24.02 ± 2.88	0.081	25.90 ± 1.21	20.76 ± 1.36	25.26 ± 1.12 **^#^	0.002	15
	(186.87)	(161.33)	(160.13)		(172.67)	(138.4)	(168.4)		
Zinc (mg)	11.51 ± 1.07	12.09 ± 1.41	12.90 ± 1.43	0.546	10.12 ± 0.36	13.22 ± 0.43 *	12.87 ± 1.47 *	0.001	15
	(76.73)	(80.60)	(86.00)		(67.47)	(88.13)	(85.8)		
Selenium (μg)	46.93 ± 3.67	60.51 ± 8.93	64.28 ± 3.52	0.053	25.31 ± 0.49	40.52 ± 2.06 *	61.70 ± 7.17 **^#^	0.000	50
	(93.86)	(121.02)	(128.56)		(50.62)	(81.04)	(123.4)		
Cholesterol (mg)	486.26 ± 58.29	498.69 ± 45.93	509.72 ± 107.69	0.916	191.53 ± 29.71	247.82 ± 54.93	451.27 ± 25.03 **^##^	0.001	300
	(162.09)	(166.23)	(169.91)		(63.84)	(82.61)	(150.42)		

* *p* < 0.05: *vs.* 1986–1988; ** *p* < 0.01: *vs.* 1986–1988; ^#^ *p* < 0.05: *vs.* 2000–2004; ^##^ *p* < 0.01: *vs.* 2000–2004. Abbreviation: RNI: recommended nutrient intake; AI: adequate intake.

Energy from fat also slightly increased among urban residents to higher than the RNI. However, the proportion of fat from animals showed a decreasing trend (from 48.12% for 2000–2004 to 37.85% for 2008–2011) (Figure 2). For rural residents, there was an obvious increase of the proportion of energy from fat overall and fat from animals specifically (Figure 2). Thus, the energy and food composition patterns are now more similar between urban and rural areas.

**Table 4:** The proportion of energy from different dietary food groups for urban and rural residents in Tianjin in three different periods (%).

Food Category	Urban			Rural		
	1986–1988	2000–2004	2008–2011	1986–1988	2000–2004	2008–2011
Cereals and grain products	48.61	37.54	41.90	68.68	74.53	48.73
Beans and their products	2.24	2.02	3.35	1.12	1.48	3.28
Potatoes and starches	1.16	1.45	1.59	1.05	0.10	1.48
Animal foods	16.79	24.54	20.73	5.18	7.74	16.56
Pure energy foods	16.51	16.19	15.24	17.27	12.04	15.92
Others	14.68	18.26	17.19	6.70	4.10	14.03
Total	100.00	100.00	100.00	100.00	100.00	100.00

## 4. Discussion

After the fourth, or reform period, from 1979 to 1985, China experienced remarkable economic progress [4]. There was a significant increase in food production capacity and food yield. Living standards and food accessibility per capita increased rapidly. The current period (since 1985) is the fifth period. Our study included data only from the fifth period, in which there was further economic improvement. The total gross domestic product (GDP) increased from 201.7 billion USD in 1980 (the fourth period) to 354.6 in 1990, 1079.9 in 2000 and 5685.5 in 2010 (the fifth period). The per capita GDP was 309, 343, 945 and 4387 USD respectively in these years [20]. The three periods (1986–1988, 2000–2004 and 2008–2011) in this study are just around these years. Fortunately, we observed many dietary changes which indicated a slight and significant improvement in the pattern of food intake in both urban and rural areas.

During the period of 1986 to 1988, food intake of key food types (cereals, vegetables, animal foods) in urban areas was abundant except for fruits, beans, milk, and the oil. In particular, animal food was very popular. However, the intake of most key food types (fruits, animal foods) in rural areas was extremely rare except for cereals. Lack of calcium (due to a lack of milk and beans) and dietary fiber (due to choice of cereals and inadequate vegetables and fruits) became a problem for both urban and rural residents. Moreover, lack of riboflavin (due to choices in vegetable types or a lack of animal foods), ascorbic acid (due to a lack of fresh fruits) and selenium (due to a lack of animal foods) were found particularly in rural areas. After this period, the first food-based dietary guideline (FBDG) was established in China in 1989, and a FGP was established in association with a revision of the FBDG in 1997 [11].

To solve the problem in the first period, the recommendation in 1997 to consume “appropriate amounts” of animal food, “plenty” of vegetables and fruits, and consume milk, beans or dairy- or bean-products “everyday” were hoped to benefit both ends of the population [11]. During the period of 2000 to 2004, although rural residents consumed a little more animal food, urban residents did not change their total amount of animal foods. There was just a slight change in the proportion of animal food (an increase in meat, poultry, milk and their products, a decrease in seafood, egg and their products). In addition, an evident decrease in cereal intake and an increase in the intake of vegetables and fruits were observed in urban areas. Cereals were no longer the main source of energy. About 50% of the energy came from protein and fat, and most of this was from animal foods. Meanwhile, there was also an obvious change in rural areas, especially the consumption of fruit (9.32-fold increase) and milk (54.38-fold increase). However, lack of calcium and dietary fiber was still a major problem. In addition, lack of retinol (due to choices in vegetable or animal foods types) became a new problem for both urban and rural residents. Furthermore, lack of energy (due to low in cereals intake) and thiamine (due to choice of refined grains) in urban areas and lack of riboflavin (due to low in animal foods intake) in rural areas were found. These changes indicated that the guide partly affected consumption. Moreover, a study showed the awareness rate of the FBDG among Chinese adults increased from 31.9% to 99.2% after education [21]. Therefore, secular and regular education should be effective to increase the awareness and provide people a scientific and reasonable guidance.

During the second period, consumption of large amounts of meat, poultry, and oils and small amount of cereals resulted in a rapid increase in the prevalence of non-communicable disease. By 2004, nearly one-fourth of all Chinese adults were overweight. Moreover, the rate of change in the Chinese overweight status was one of the most rapid in the world, far larger than that in the United States [22,23], and these changes are accelerating [24]. There is in China a large increase in nutrition-related causes of death, such as cancer and cardiovascular disease [25,26]. These causes are directly linked to diet, activity, and obesity [27,28]. Therefore, basic, scientific guideline on a balanced diet is needed for the Chinese population.

Before the third period in this study, a further revision of the FBDGs and the FGP took place in 2007. In China, most of the studies on dietary changes were before the third period [2,10,19]. They found a shift toward a high-fat, high-energy-density and low-fiber diet in the Chinese nutrition transition. In this study, we added some new findings after 2007. We found an increase in the consumption of cereals and in the intake of dietary fiber and thiamine during the period of 2008 to 2011, although the energy intake from carbohydrates was only 51%. This might related to the guidelines which added “including appropriate amount of coarse grains” and the improved awareness of healthy diet among Chinese adults. We also found an increase in bean consumption and a decrease in meat consumption in urban areas. In the meantime, a sharp increase in the consumption of fruits and beans products was found in rural areas. Nevertheless, intake of cholesterol for both urban and rural residents remarkably exceeded 300 mg/day. Furthermore, intakes of every kind of animal food and edible oil also increased significantly for rural residents. One of the reasons for the consumption of a large amount of edible oils may be that both the importation of soybean oil and that domestic production skyrocketed [29,30].

Additionally, the intake of energy and various nutrients for both urban and rural areas can reach 80% RNI/AI or above, but the intake of calcium, retinol and dietary fiber was still not up to the 80% RNI/AI though they increased more than the previous period. A higher intake of iron was also found in this study. This might be partially due to a higher intake of animal food and can explain the lower prevalence (4.6%) of anemia in Tianjin [31]. However, the iron intake was much higher than RNI (over 160%). The result was similar with that of the 2002 NNHS [18]. Moreover, epidemiological studies provide evidence that elevated iron stores are a risk factor for developing cardiovascular and metabolic abnormalities, such as atherosclerosis, diabetes and metabolic syndrome [32,33]. Therefore, a high iron intake in the population is of concern and needs further research. In a word, scientific and reasonable dietary guideline are very important and useful for residents.

The classic Chinese diet includes cereals and vegetables with few animal foods [34]. Many studies indicate that diets largely based on plant foods offer a number of nutritional benefits [35,36,37]. Lower consumption of meats and higher consumption of grains have long been considered to be part of a healthy diet to help reduce risks for cardiovascular disease [37,38,39,40]. However, increased meat consumption and decreased cereal consumption over time is a worldwide phenomenon, especially during recent years, among developing countries that have enjoyed rapid economic development. This dietary pattern is related to increased chronic morbidity, while a greater intake of vegetables, fruits, cereals, nuts, and beans has been independently associated with a lower risk for several chronic diseases, such as cardiovascular disease and many cancers [36,37,41]. Recent findings related to global climate change suggest that high-meat diets are less sustainable [42,43]. Although the nutritional status significantly improved, Chinese will still face the simultaneous challenges of undernutrition and overnutrition for a certain period of time.

This study had several limitations. First, the study population was only from Tianjin, China. Therefore, caution should be used when generalizing the findings from this study to broader populations with different socioeconomic status. Secondly, the third survey used 24 h recall, and the other two used weighted-food records (a gold standard method to assess dietary intakes). Because the relative differences of food and nutrients intakes were less than 10% by two methods [15], we considered data from the three surveys as comparable. Finally, dietary patterns may be affected by many other factors, such as psychosocial–behavioral, educational levels and healthy status [44]. Thus, further study is needed to improve our observation.

## 5. Conclusions

From this study, we found that there are signs of decline of meat intake in the third period, and meat consumption had peaked and began to decrease in urban areas. Although the contribution of fat to total energy intake did not decrease, fats from plant sources increased. Furthermore, after a very large decrease in cereal consumption, it began to show an increasing trend. Consumption of both beans and milk products showed a stable increase. Although the nutrition transition of rural areas was usually a period slower than urban areas, the dietary structure of rural residents was more and more close to urban residents’. During the important stage of nutrition transition, both the people’s efforts and the national efforts are necessary to improve human health.

## Supplementary Files

Supplementary File 1
