# Supplementary material for: Dietary Changes over 25 Years in Tianjin Residents: Findings from the 1986–1988, 2000–2004, and 2008–2011 Nutrition Surveys
Source: Nutrients. 2016 Jan 22;8(2):62. doi: 10.3390/nu8020062 (PMC4772026; doi:10.3390/nu8020062)
Supplement: Supplementary File 1 [file nutrients-08-00062-s001.docx]

Supplementary Materials: Dietary Changes over
25 Years in Tianjin Residents: Findings from the 1986–1988, 2000–2004, and 2008–2011 Nutrition Surveys

Xuan Wang, Yuntang Wu, Xumei Zhang, Meilin Zhang and Guowei Huang

**Table S1.** The proportion of different dietary food groups for urban and rural residents in Tianjin in three different periods (%).

| **Food Category *** | **Urban** | | | **Rural** | | |
| --- | --- | --- | --- | --- | --- | --- |
|  | **1986–1988** | **2000–2004** | **2008–2011** | **1986–1988** | **2000–2004** | **2008–2011** |
| Cereals and tubers products | 38.26 | 22.63 | 29.75 | 59.79 | 47.09 | 36.06 |
| Vegetables | 24.05 | 30.48 | 20.42 | 23.70 | 30.34 | 20.78 |
| Fruits | 4.26 | 16.99 | 15.58 | 1.01 | 8.46 | 16.29 |
| Meats and poultry and their products | 7.37 | 7.83 | 5.68 | 2.32 | 3.04 | 4.79 |
| Fish and shellfish | 5.51 | 4.04 | 3.43 | 3.11 | 2.19 | 2.82 |
| Egg & egg products | 6.39 | 4.43 | 4.63 | 3.47 | 3.64 | 4.52 |
| Milk & dairy products | 3.88 | 4.99 | 5.24 | 0.01 | 0.68 | 1.72 |
| Beans and their products | 1.87 | 1.26 | 6.94 | 1.42 | 0.96 | 4.87 |
| Oils | 3.38 | 1.96 | 2.03 | 2.73 | 2.59 | 2.50 |
| Others | 5.03 | 5.39 | 6.30 | 2.44 | 1.01 | 5.65 |
| Total | 100.00 | 100.00 | 100.00 | 100.00 | 100.00 | 100.00 |

* Food Category is classified according to Chinese food guide pagoda.

**
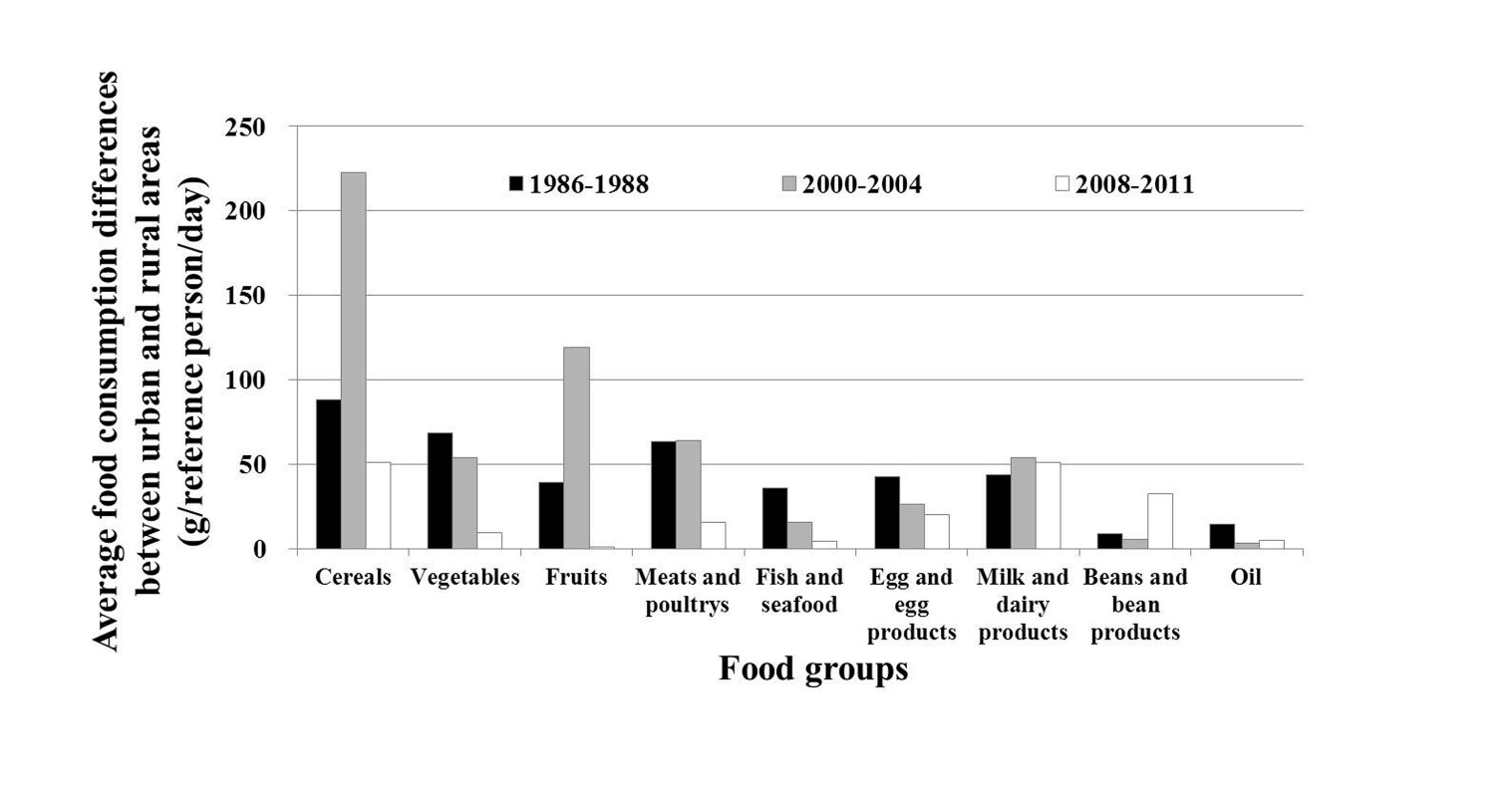
**

**Figure S1.** Average food consumption differences between urban and rural areas in three different periods. The bars represent the absolute value of differences between urban and rural areas. Difference in most of food groups were decreasing between urban and rural areas, especially in vegetables, fruits, seafood, and oil.
